# Supplementary material for: Time-dependent effect of 1,6-hexanediol on biomolecular condensates and 3D chromatin organization
Source: Genome Biol. 2021 Aug 17;22:230. doi: 10.1186/s13059-021-02455-3 (PMC8369800; doi:10.1186/s13059-021-02455-3)
Supplement: Supplementary file 1 — Additional file 1: Fig. S1. Controls of 1,6-HD treatment and effects of 1,6-HD treatment on cell viability, nuclear volume, chromatin mobility and chromatin condensation. Fig. S2 1.5%, 2 min 1,6-HD treatment dissolved biomolecular condensates in NPCs and MEFs without affecting cell viability and nuclear volume. Fig. S3 Hi-C reproducibility across replicates and with published data. Fig. S4 Extended analysis of compartmental changes and features of strengthened or weakened compartment loci after short-term 1,6-HD treatment. Fig. S5 Features of compartment loci in different PC1 range and homogeneities of B-B interactions. Fig. S6 Analysis of compartment switch and features of switched compartment loci. Fig. S7 Extended analysis and validation of TAD changes and features of reorganized TAD boundaries after short-term 1,6-HD treatment. Fig. S8 Comparison between condensate-component-enriched interactions and E-P interactions and clues for redundancy between condensate components. [file 13059_2021_2455_MOESM1_ESM.pdf]

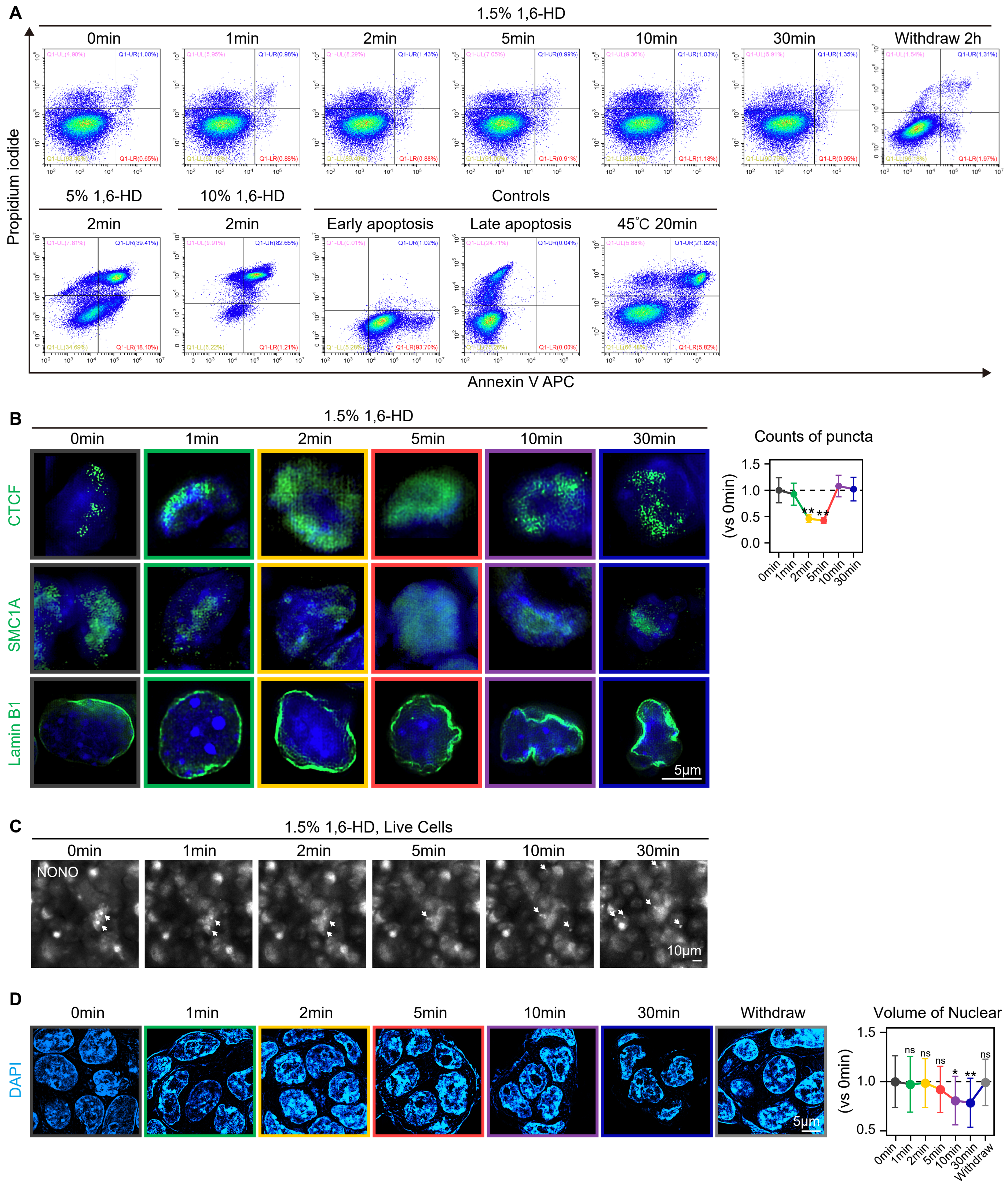

Fig. S1 (part 1 of 2)

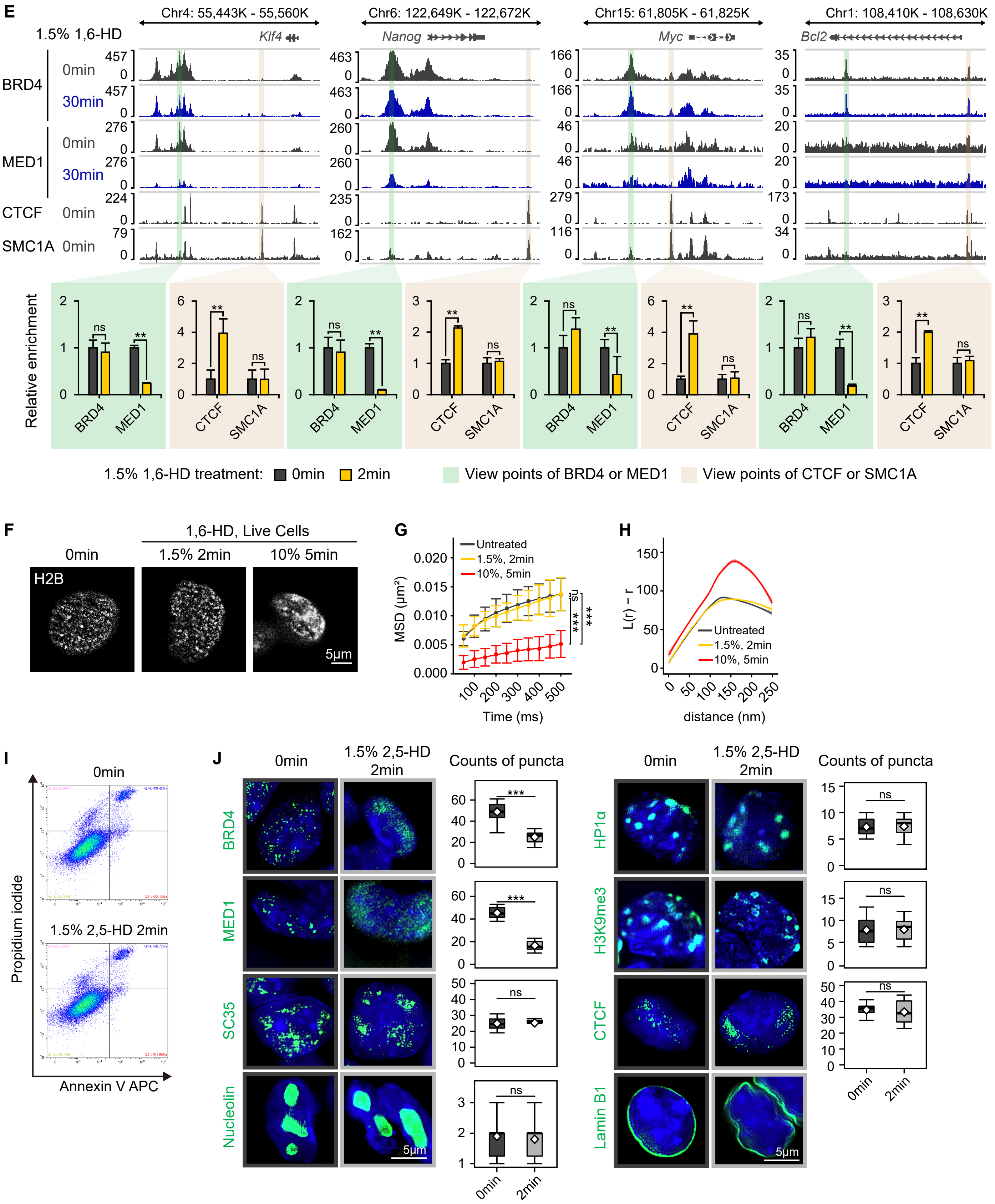

Fig. S1 (part 2 of 2) (See legend on next page.)

Fig. S1 Controls of 1,6-HD treatment and effects of 1,6-HD treatment on cell viability, nuclear volume, chromatin mobility and chromatin condensation. **A** Apoptosis of mouse embryonic stem cells (mESCs) upon 1,6-HD treatment. **B** Left: Structured illumination microscopy (SIM) images of IF for the proteins indicated by green words. IF for indicated protein is colored green, and signal from DAPI is colored dark blue. Right: Counts of IF puncta within nucleus at each time points versus 0min. Error bars represent standard deviation (SD) from 30 cells each. All p values were determined using the Student's t test. **C** Live-cell fluorescence microscopy of endogenous tagged paraspeckles (indicated by NONO). The white arrows indicate observed condensates. **D** Left: SIM images of DAPI-stained mESCs upon 1,6-HD treatment at indicated time points. Right: quantification of nuclear volume versus 0min. For each condition, 30 cells were used for quantification of nuclear volume. All p values were determined by the Student's t test. **E** Top: genome browser view of published ChIP-seq data. The y axis shows reads per kilobase per million reads (RPKM). Bottom: Relative enrichment of indicated factors determined by ChIP-qPCR assay at indicated sites. All p-values were determined by the Student's t test. Three biological replicates were assayed in ChIP-qPCR assays. **F** Super-resolution live-cell imaging of stably-expressed H2B-GFP. **G** Mean square displacement plots indicating chromatin motion (± SD among cells). All p values were determined using the Kolmogorov-Smirnov test. **H** L-function plot indicating chromatin condensation. **I** Apoptosis analysis of mESCs upon 2,5-HD treatment. **J** Left: SIM images of IF for the proteins indicated by green words. IF for indicated protein is colored green, and signal from DAPI is colored dark blue. Right: Counts of IF puncta within nucleus at indicated time points. For each type of condensate under different condition, 30 cells were used for quantification of puncta. All p values were determined using the Wilcoxon rank-sum test.

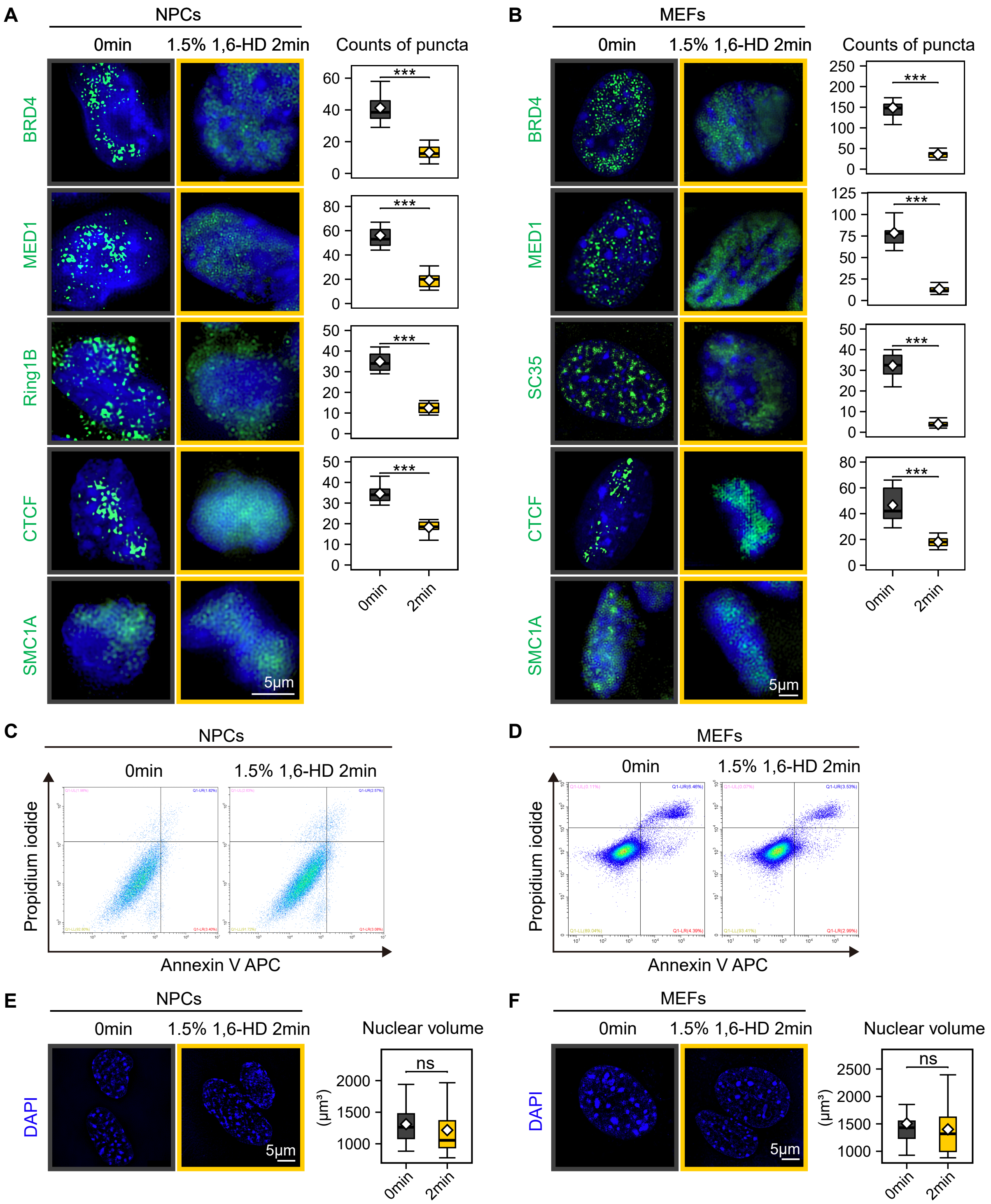

Fig. S2 1.5%, 2min 1,6-HD treatment dissolved biomolecular condensates in NPCs and MEFs without affecting cell viability and nuclear volume. **A**, **B** Left: SIM images of IF for the proteins indicated by green words in NPCs (**A**) or MEFs (**B**). IF for indicated protein is colored green, and signal from DAPI is colored dark blue. Right: Counts of IF puncta within nucleus at each time points. For each type of condensate under different condition, 30 cells were used for quantification of puncta. All p values were determined using the Wilcoxon rank-sum test. **C**, **D** Apoptosis of NPCs (**C**) or MEFs (**D**) upon 1,6-HD treatment. **E**, **F** Left: SIM images of DAPI-stained NPCs (**E**) or MEFs (**F**). Right: quantification of nuclear volume. For each cell type under different condition, 30 cells were used for quantification of nuclear volume. All p values were determined using the Wilcoxon rank-sum test.

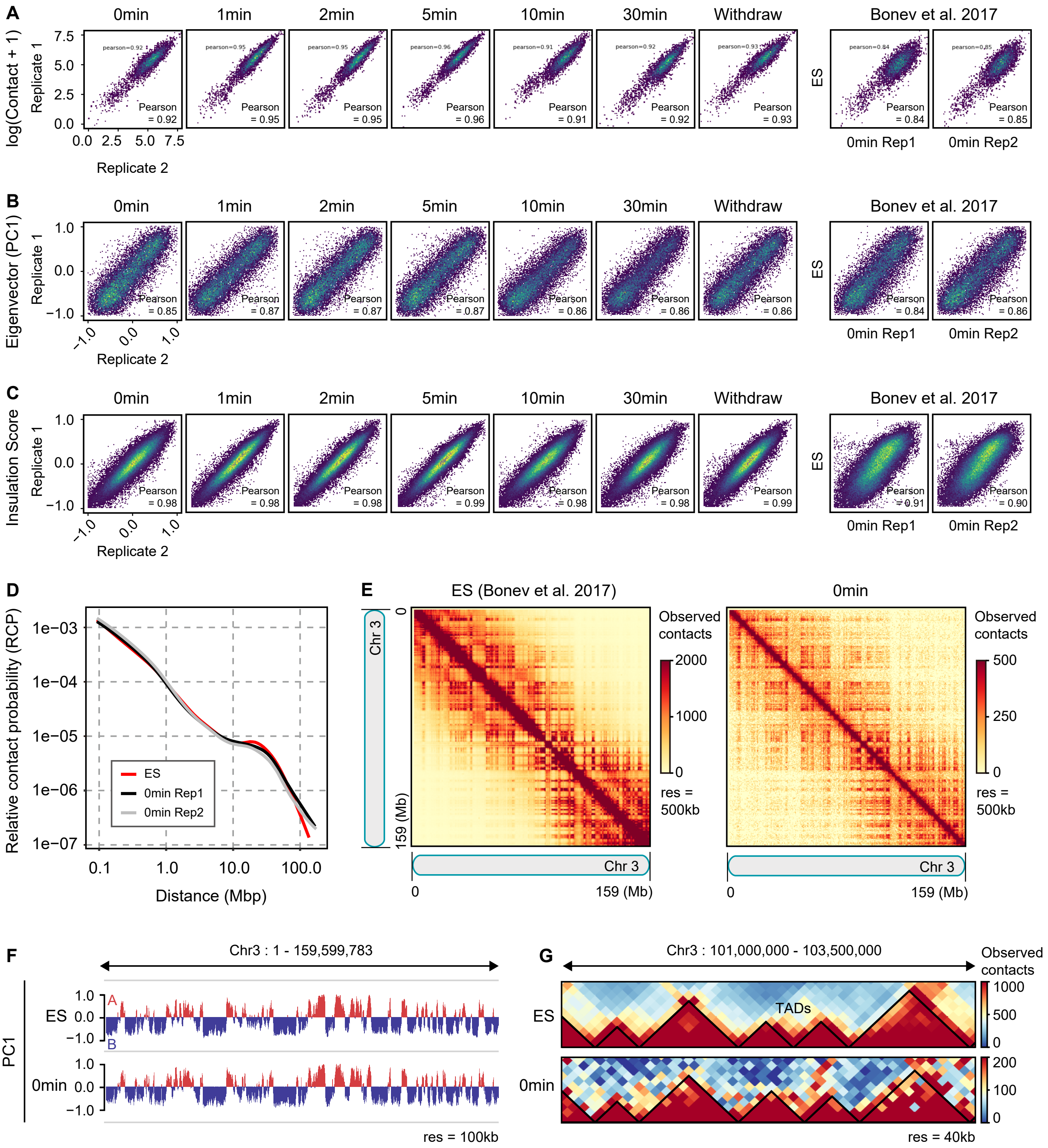

Fig. S3 Hi-C reproducibility across replicates and with published data. **A** Pairwise correlations of contact matrices between the two replicates contact matrices at indicated time point and between 0min and published mES Hi-C data. **B** Pairwise correlations of eigenvectors (PC1) at 100kb resolution. **C** Pairwise correlation of insulation scores at 40kb resolution. **D** Relative contact probability (RCP) at 40kb resolution. **E** Observed contact matrices for Chr3 at 500kb resolution. **F** PC1 across Chr3 at 100kb resolution. Red part refers to A compartment and blue part refers to B compartment. **G** TADs for Chr3: 101-103.5Mb at 40kb resolution.

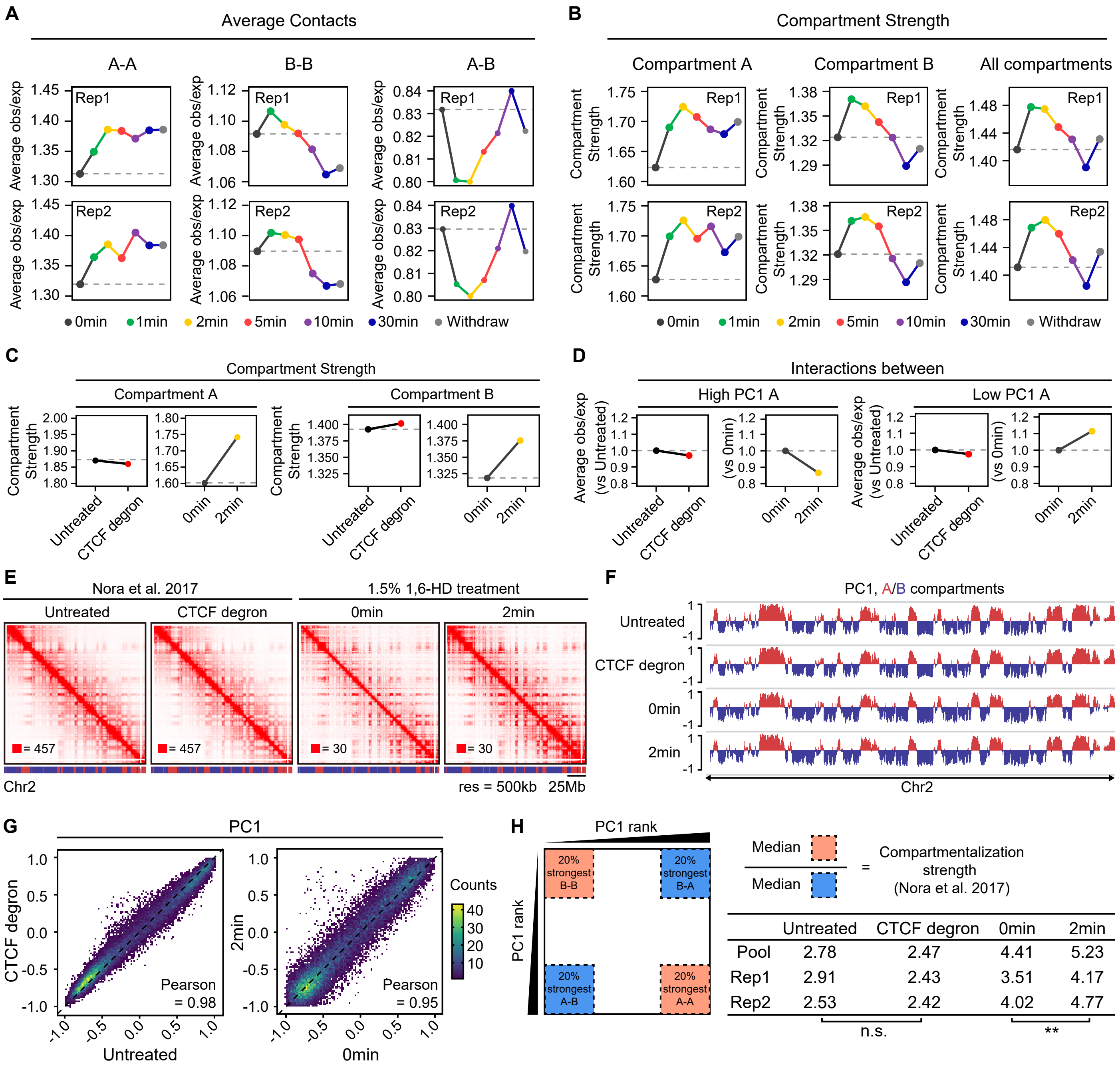

Fig. S4 (part 1 of 2)

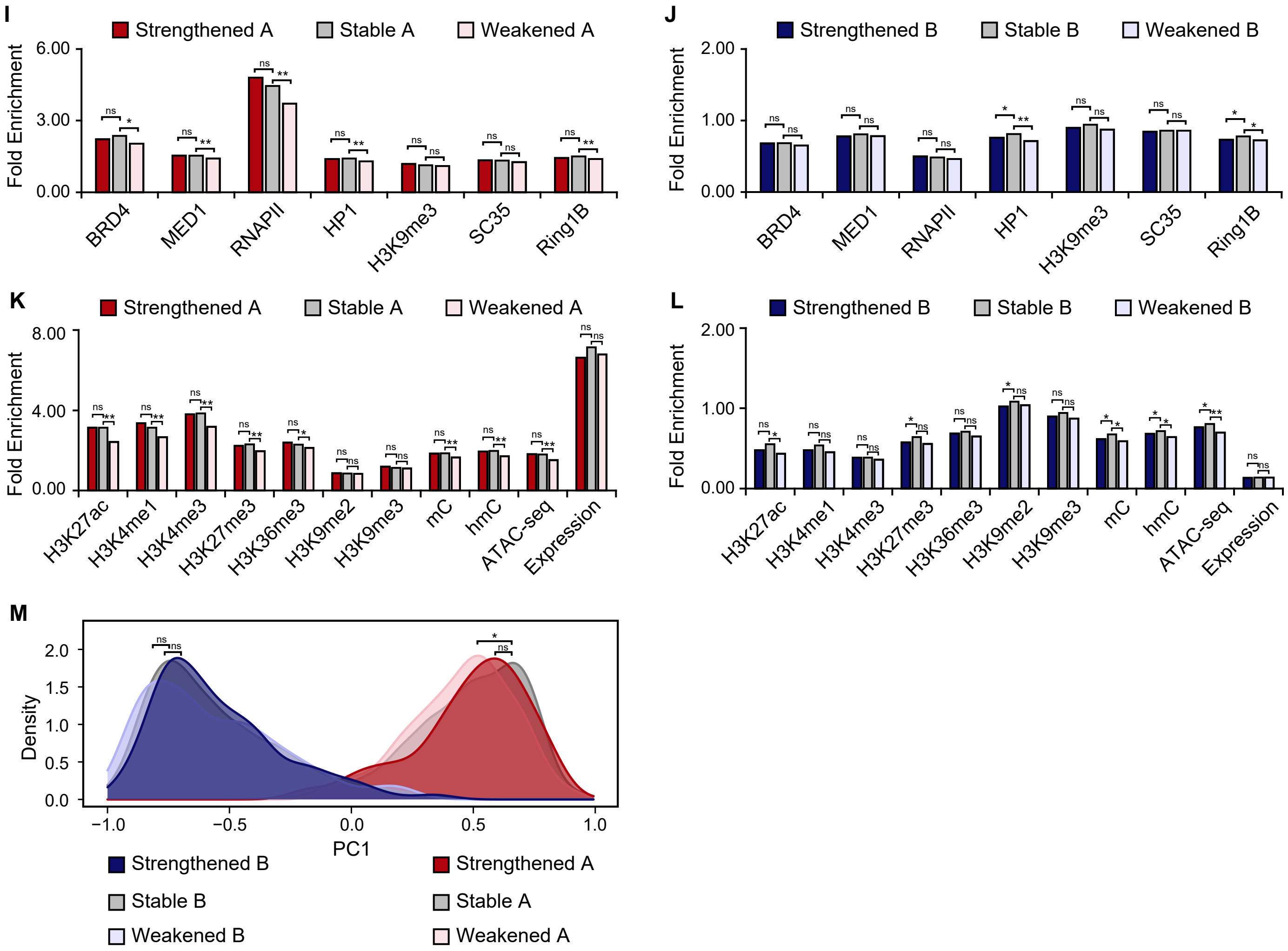

Fig. S4 (part 2 of 2) Extended analysis of compartmental changes and features of strengthened or weakened compartment loci after short-term 1,6-HD treatment. **A** Average contacts between 100kb bins from the same (A-A, B-B) and different (A-B) compartment types at indicated time point upon 1,6-HD treatment in different biological replicates. **B** Compartment strength of all 100kb bins from A-, B- and both compartment types at indicated time point upon 1,6-HD treatment in different biological replicates. **C** Comparison of compartment strength (100kb resolution) between CTCF degradation and 1,6-HD treatment. “Untreated” refers to untreated CTCF-AID mESCs and “CTCF degron” refers to mESCs with auxin-mediated degradation of CTCF. **D** Average interactions between strong A-featured loci (high PC1 A, PC1> 0.8) or weak A-featured loci versus untreated cells upon CTCF degradation or 1,6-HD treatment. **E** Contact maps at across Chr2. Bars below denotes compartment A (red) or B (blue) at 100kb resolution. **F** PC1 across Chr2 at 100kb resolution. Red part refers to A compartment and blue part refers to B compartment. **G** Pairwise correlations of PC1 at 100kb resolution. **H** Quantification of compartmentalization strength as Nora et al. 2017. P values were determined by the paired t-test. **I**, **J** Fold enrichment of ChIP-seq signal of indicated condensate-components. All p-values were determined by the Wilcoxon rank-sum test. **K**, **L** Fold enrichment of ChIP-seq signal of key histone markers, ATAC-seq signal indicating chromatin accessibility and expression. All p-values were determined by the Wilcoxon rank-sum test. **M** Density plot of PC1 distribution.

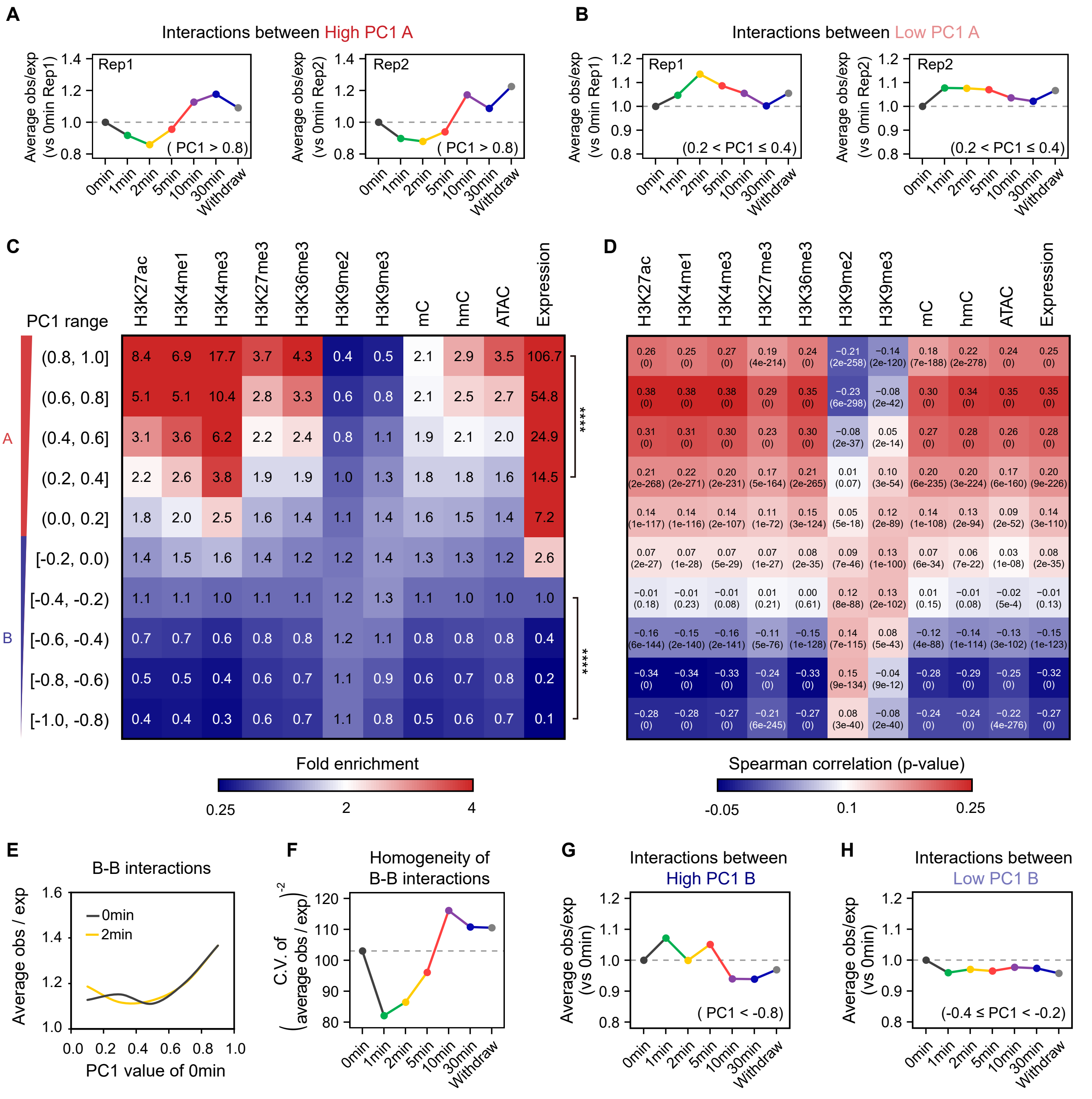

Fig. S5 Features of compartment loci in different PC1 range and homogeneities of B-B interactions. **A**, **B** Average interactions between strong A-featured loci (high PC1 A, PC1> 0.8) or weak A-featured loci versus untreated cells in different biological replicates. **C** Fold enrichment of ChIP-seq signal of key histone markers, ATAC-seq signal indicating chromatin accessibility and expression. The rightest square brackets indicate that all features in the two rows are significantly different. All p-values were determined by the Wilcoxon rank-sum test. **D** Spearman correlation coefficients (p values in parentheses) for key histone markers, chromatin accessibility and expression with compartment loci in different PC1 range. All p-values were determined by the Wilcoxon rank-sum test. **E** Average B-B interactions of loci in different PC1 range. **F** Homogeneity of B-B interactions at each time point. **G** Average interactions between strong B-featured loci (high PC1 B, PC1< -0.8) versus 0min at each time point. **H** Average interactions between weak B-featured loci (low PC1 B -0.4 ≤ PC1 < -0.2) versus 0min at each time point.

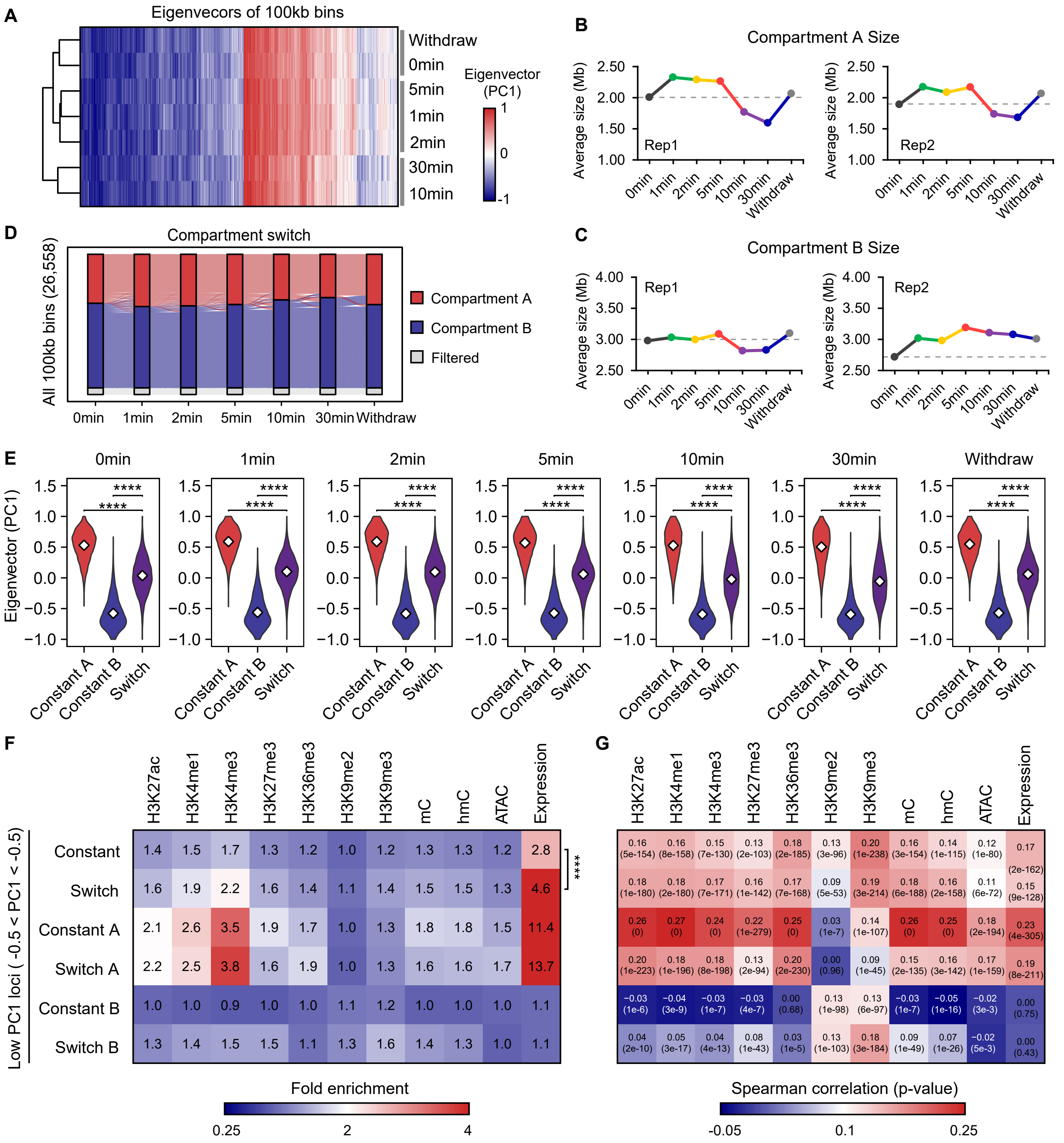

Fig. S6 Analysis of compartment switch and features of switched compartment loci. **A** Clustering of PC1 of 100kb bins by ward's method. **B**, **C** Average size of compartment A or B regions at each time point in different biological replicates. **D** Alluvial plot of compartment switch. **E** PC1 of constant compartment A loci, constant compartment B loci, and switched compartment loci at each time point. All p-values were determined by the Wilcoxon rank-sum test. **F** Fold enrichment of ChIP-seq signal of key histone markers, chromatin accessibility and expression. The rightest square brackets indicate that all features in the two rows are significantly different. **G** Spearman correlation coefficients (p values in parentheses) for key histone markers, chromatin accessibility and expression with loci of interest. All p-values were determined by the Wilcoxon rank-sum test.

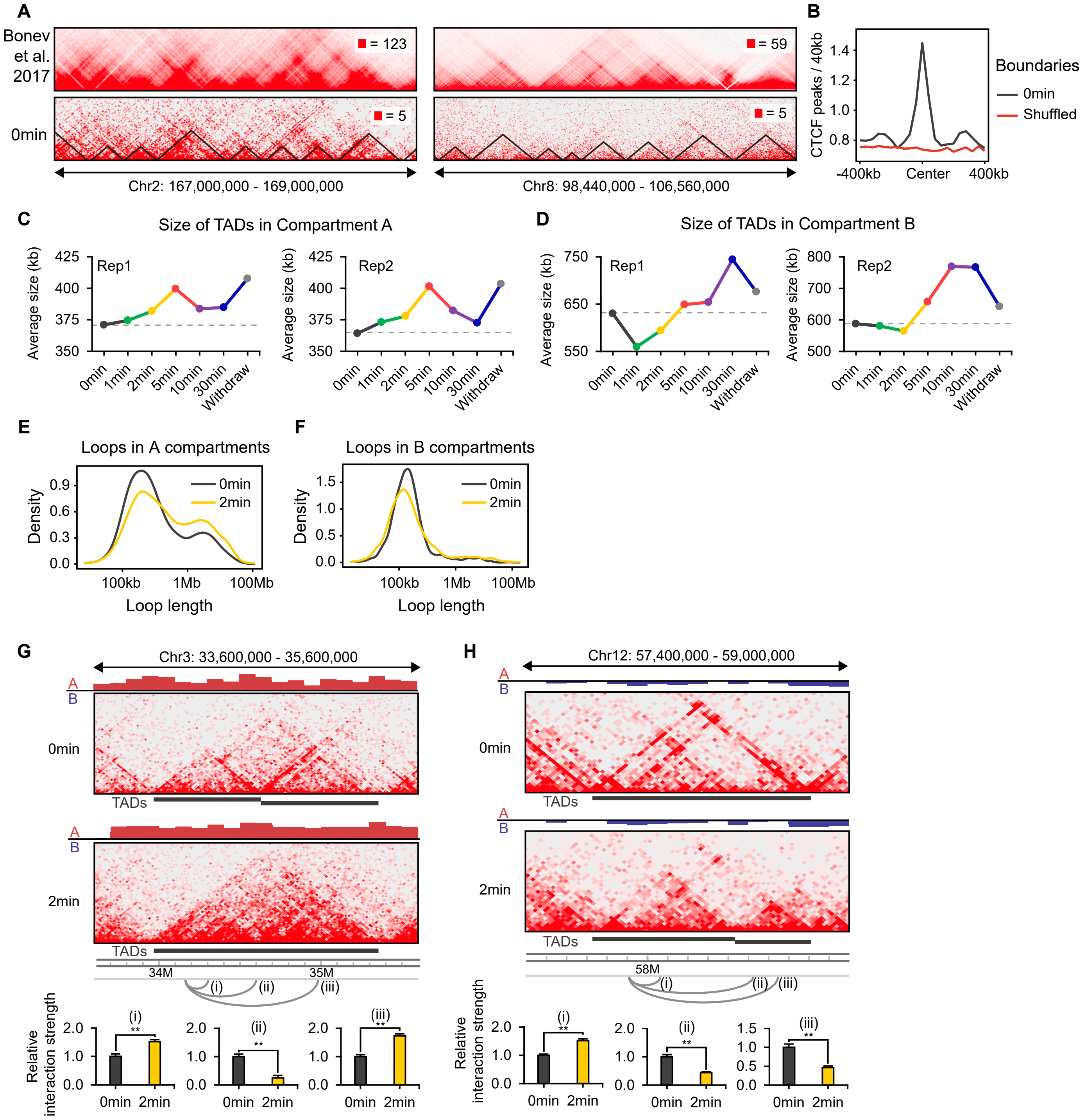

Fig. S7 (part 1 of 2)

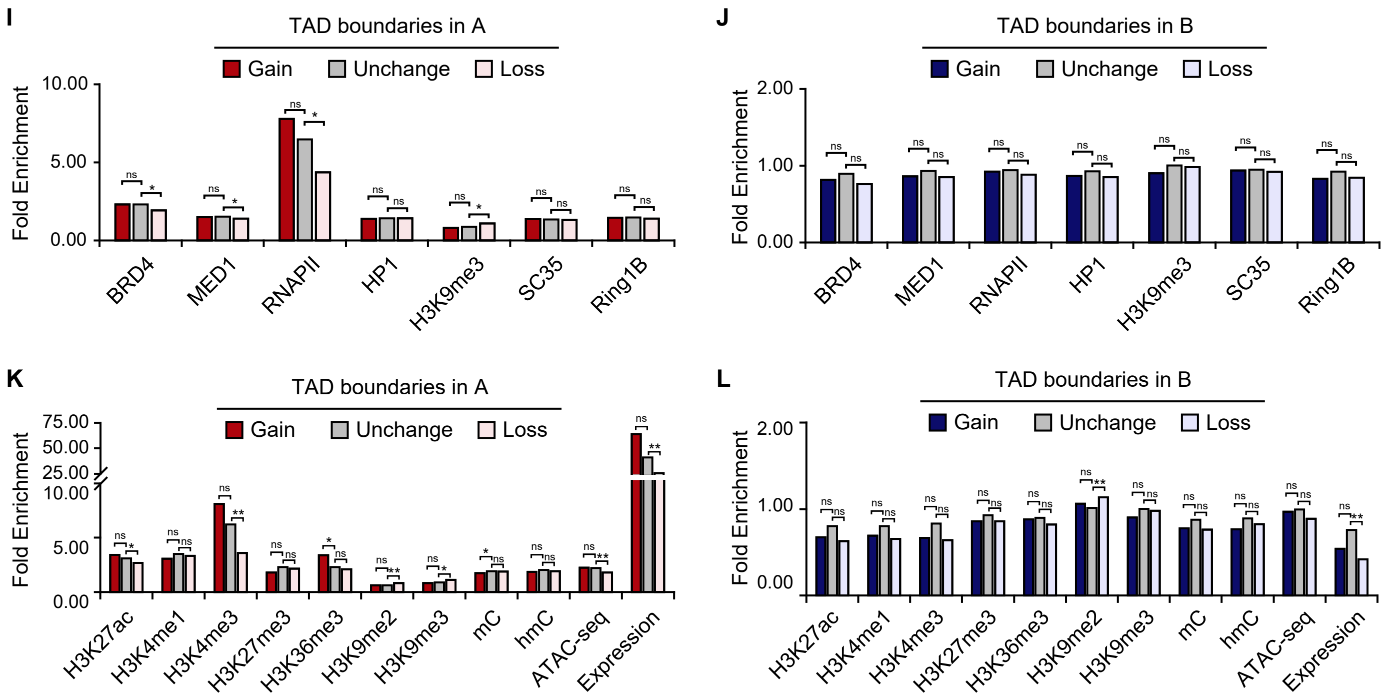

Fig. S7 (part 2 of 2) Extended analysis and validation of TAD changes and features of reorganized TAD boundaries after short-term 1,6-HD treatment. **A** Contact maps of published ES Hi-C data and data of 0min. TADs are indicated by black lines. **B** Density of CTCF ChIP-seq peaks in identified TAD boundaries compared with shuffled regions. **C**, **D** Average size of TADs in compartment A or compartment B at each time point in different biological replicates. **E**, **F** Density plot showing the length distribution of the chromatin loops. **G**, **H** Top: PC1 indicates compartment assignment and contact maps at indicated time point. Identified TADs are indicated by thick black lines. Bottom: fold change of strength of indicated interactions determined by 3C-qPCR assay. All p-values were determined by the Student's t test. Three biological replicates were assayed for 3C-qPCR experiment. **I**, **J** Fold enrichment of ChIP-seq signal of condensate-components. All p-values were determined by the Wilcoxon rank-sum test. **K**, **L** Fold enrichment of ChIP-seq signal of key histone markers, chromatin accessibility and expression. All p-values were determined by the Wilcoxon rank-sum test.

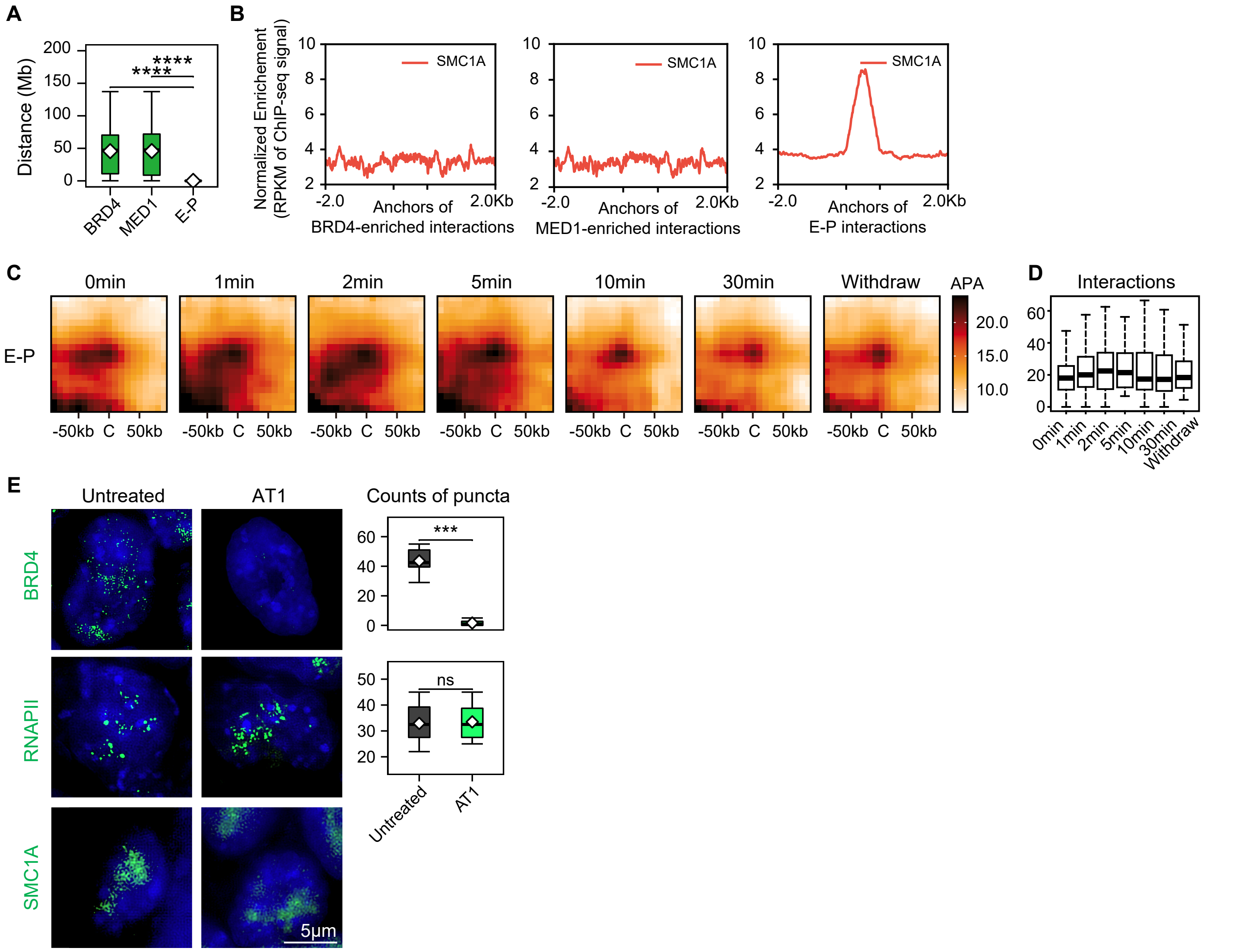

Fig. S8 Comparison between condensate-component-enriched interactions and E-P interactions and clues for redundancy between condensate components. **A** Distance of BRD4- or MED1-enriched interactions and Enhancer-Promoter interactions. **B** Cohesin enrichment on anchors of indicated interactions. The y axis shows RPKM. **C**, **D** Left: APA analysis of Enhancer-Promoter (E-P) interactions at 10kb resolution. E-P interactions were identified using data in GSE101498. Right: Average interactions surrounding the center (<30kb) of structural factor-mediated interactions. Pairwise Wilcoxon rank-sum test was performed but no significant changes were detected. **E** Left: SIM images of IF for the proteins indicated by green words. IF for indicated protein is colored green, and signal from DAPI is colored dark blue. Right: Counts of IF puncta within nucleus under different treatments. All p values were determined using the Wilcoxon rank-sum test. For each type of condensate under different condition, 30 cells were used for quantification of puncta. All p values were determined using the Wilcoxon rank-sum test.
